# Immuno-PET Imaging of the Programmed Cell Death-1 Ligand (PD-L1) Using a Zirconium-89 Labeled Therapeutic Antibody, Avelumab

**DOI:** 10.1177/1536012119829986

**Published:** 2019-05-02

**Authors:** Elaine M. Jagoda, Olga Vasalatiy, Falguni Basuli, Ana Christina L. Opina, Mark R. Williams, Karen Wong, Kelly C. Lane, Steve Adler, Anita Thein Ton, Lawrence P. Szajek, Biying Xu, Donna Butcher, Elijah F. Edmondson, Rolf E. Swenson, John Greiner, James Gulley, Janet Eary, Peter L. Choyke

**Affiliations:** 1 Molecular Imaging Program, National Cancer Institute, Bethesda, MD, USA; 2 Imaging Probe Development Center, National Heart, Lung, and Blood Institute, National Institutes of Health, Rockville, MD, USA; 3 PET Department, Clinical Center, NIH, Bethesda, MD, USA; 4 Pathology & Histotechnology Lab Frederick National Laboratory for Cancer Research, NCI, Frederick, MD, USA; 5 Laboratory of Tumor Immunology and Biology, National Cancer Institute, Bethesda, MD, USA; 6 Genitourinary Malignancies Branch, National Cancer Institute, Bethesda, MD, USA; 7 Clinical Research Directorate/CMRP, Leidos Biomedical Research Inc. (formerly SAIC-Frederick, Inc.), Frederick National Laboratory for Cancer Research, Frederick, MD, USA; 8 Cancer Imaging Program, National Cancer Institute, Bethesda, MD, USA

**Keywords:** immuno-PET imaging, PD-L1, anti-PD-L1 antibodies, avelumab, [^89^Zr]Zr-DFO-PD-L1 mAb

## Abstract

**Objective:**

The goal is to evaluate avelumab, an anti-PD-L1 monoclonal immunoglobulin G antibody labeled with zirconium-89 in human PD-L1-expressing cancer cells and mouse xenografts for clinical translation.

**Methods:**

[^89^Zr]Zr-DFO-PD-L1 monoclonal antibody (mAb) was synthesized using avelumab conjugated to desferrioxamine. In vitro binding studies and biodistribution studies were performed with PD-L1+MDA-MB231 cells and MDA-MB231 xenograft mouse models, respectively. Biodistributions were determined at 1, 2, 3, 5, and 7 days post coinjection of [^89^Zr]Zr-DFO-PD-L1 mAb without or with unlabeled avelumab (10, 20, 40, and 400 µg).

**Results:**

[^89^Zr]Zr-DFO-PD-L1 mAb exhibited high affinity (K_d_ ∼ 0.3 nM) and detected moderate PD-L1 expression levels in MDA-MB231 cells. The spleen and lymph nodes exhibited the highest [^89^Zr]Zr-DFO-PD-L1 mAb uptakes in all time points, while MDA-MB231 tumor uptakes were lower but highly retained. In the unlabeled avelumab dose escalation studies, spleen tissue–muscle ratios decreased in a dose-dependent manner indicating specific [^89^Zr]Zr-DFO-PD-L1 mAb binding to PD-L1. In contrast, lymph node and tumor tissue–muscle ratios increased 4- to 5-fold at 20 and 40 µg avelumab doses.

**Conclusions:**

[^89^Zr]Zr-DFO-PD-L1 mAb exhibited specific and high affinity for PD-L1 in vitro and had target tissue uptakes correlating with PD-L1 expression levels in vivo. [^89^Zr]Zr-DFO-PD-L1 mAb uptake in PD-L1+tumors increased with escalating doses of avelumab.

## Introduction

The programmed cell death-1 receptor (PD-1)/ programmed cell death ligand 1 (PD-L1) pathway has emerged as an important immunotherapeutic target for cancer.^1,2^ The PD-1/PD-L1(B7-H1) pathway provides “immune checkpoint” regulation and when activated results in the inhibition and exhaustion of cytotoxic T-cell responses, thereby providing a regulatory mechanism in normal tissues.^3^ Recent research has shown that tumors utilize this pathway to create an immunosuppressive microenvironment that benefits tumor progression.^4,5^ The pathway comprised of the cell surface PD-1 receptor and its 2 ligands (PD-L1 and PD-L2) is activated upon the PD-1 receptor binding to its ligand which in turn signals the inactivation of cytotoxic T-cells.^6^ The PD-1 receptor is expressed on various immune cells (T and B cells, dendritic cells, monocytes and macrophages), whereas PD-L1 expression occurs on hematopoietic cells and non-hematopoietic cells and tissues (endothelial and epithelial cells, heart, vascular endothelium, pancreas, liver, lung, and skin).^1,7^ PD-L1 expression is normally constitutive but will be upregulated in response to infection or inflammation by proinflammatory cytokines such as γ-interferon.^1,8^ Cancer cells take advantage of this coinhibitory immune regulation by overexpressing PD-L1, thus escaping immune detection. Overexpression of PD-L1 has been observed across a wide variety of human cancers including skin, blood, lung, breast, ovarian, gastric, and prostate and is linked to a worse prognosis.^6,9,10^ Therapeutics designed to block PD-1 receptor binding to its respective ligand have been shown to be beneficial in reversing the immunosuppressive environment.^11,12^

To date, several monoclonal antibodies (mAb) targeting the PD-1/PD-L1 pathway have been approved by the Food and Drug Administration (FDA) including nivolumab and pembrolizumab.^13^ In these clinical trials, durable responses were observed but only in a subset of patients who were not all identified as positive for PD-L1 expression using immunohistochemistry (IHC) from tumor biopsy tissue.^14^ Although tumor expression of PD-L1 determined from IHC methods has shown promise as a predictive biomarker for patient selection and therapeutic responses, these IHC results have not been conclusive. For instance, some responders were scored as PD-L1 negative, and some nonresponders were scored as PD-L1 positive.^15,16^ This may relate to differences between in vivo “real-time” expression compared to IHC results from an earlier biopsy specimen. Additional issues include differences in detection levels of the PD-L1 mAb used for the IHC and the inherent sampling limitations of biopsy specimens. Moreover, most studies score tumor PD-L1 expression and may not include the expression on other cells in the tumor microenvironment which may be an important factor in determining therapeutic responsiveness.^14,17-19^

Radiolabeling of these antibodies could provide in vivo expression levels of PD-L1 in tumors which could help select patients for this therapy. While the high affinity and specificity of these therapeutic molecules readily fulfill a primary requirement for a successful positron emission tomography (PET) imaging agent, these large molecular weight molecules require longer lived PET radionuclides to account for the slower clearance from non-target tissue. Atezolizumab is one of the new therapeutic anti-PD-L1 mAb, which has been radiolabeled with copper-64 (t_1/2_ = 12.7 hours) and was shown to have potential for PD-L1 immuno-PET imaging in preclinical human tumor xenograft mouse models in which tumor uptakes of copper-64 labeled atezolizumab were consistent with known tumor PD-L1 expression levels.^20^ Although these tumors had high uptake levels and were discernible in biodistribution and imaging studies, respectively, some nontarget tissue uptake was seen as well. These studies would suggest that radiolabeling the mAb with a longer-lived radionuclide (>12.7 hours) would make possible imaging at later times, thereby achieving higher tumor to background ratios due to increased clearance.

In this report, avelumab, a therapeutic anti-PD-L1 monoclonal immunoglobulin G (IgG) antibody (PD-L1 mAb) was labeled with the longer-lived radionuclide, zirconium-89 (t_1/2_= 78.4 hours) and evaluated in vitro and in vivo in PD-L1 expressing human tumor cells and mouse xenograft models, respectively. In vivo biodistribution evaluations with escalating doses of unlabeled mAb were also included to determine whether tumor uptake may be improved with prior specific mAb dose loading which was previously reported with indium-111 radiolabeled atezolizumab. This fully humanized mAb recognizes both human and murine PD-L1 with high affinity and functions not only as an immune checkpoint inhibitor but also as a mediator of antibody-dependent cellular toxicity (ADCC).^21^ Avelumab has shown efficacy in preclinical mouse bladder cancer models and is currently in human clinical trials in which positive patient responses were observed but not reliably predicted by PD-L1 IHC.^12,17,22,23^ This work represents a prelude to potential clinical PET imaging with zirconium-89 avelumab which may establish the relevance of tumor PD-L1 expression as a biomarker for patient selection and identifying early therapeutic responses. The overall goal from these preclinical studies was to collect the in vitro binding data and the human dosimetry estimates of zirconium-89 labeled avelumab required for an IND filing for clinical application.

## Materials and Methods

### Cell Lines and Reagents

PD-L1 mAb (avelumab [human IgG1]; EMD-Serono [Rockland, Massachusetts]) was provided by Dr John Greiner (NCI, Bethesda, Maryland). The *p*-isothiocyanatobenzoyl- desferrioxamine (DFO-Bz-NCS) was purchased from Macrocyclics, Inc. (Plano, Texas). Sodium acetate and Tris-HCl were purchased from Thermo Fisher Scientific (Waltham, Massachusetts). Whole human serum was obtained from MP Biomedicals, LLC (Solon, Ohio). All other chemicals and solvents were received from Sigma Aldrich (St Louis, Missouri) and used without further purification. PD-10 desalting columns were obtained from GE Healthcare Biosciences (Pittsburgh, Pennsylvania). Zirconium-89 oxalate was obtained from the National Institutes of Health cyclotron facility (Bethesda, Maryland). Instant thin-layer chromatography (iTLC) papers were purchased from Biodex Medical Systems, Inc. (Shirley, New York). The iTLC papers were developed using 20 mmol/L citric buffer (pH 5) and read in an Eckert & Ziegler TLC scanner (B-AR2000 -1; Eckert & Ziegler, Hopkimton, Massachusetts). Analytical high-performance liquid chromatography (HPLC) analyses were performed on an Agilent 1200 Series instrument equipped with a multi-wavelength UV detector connected in series with a Bioscan flow count radiodetector. The size-exclusion column (SE, 4.6 mm ID × 30 cm, 4 µm), TSKgel SuperSW3000, was obtained from Tosoh Bioscience LLC (King of Prussia, Pennsylvania). HPLC condition: eluent, 0.1 mol/L sodium phosphate, 0.1 mol/L sodium sulfate, 0.05% sodium azide, 10% *iso*-propyl alcohol (pH 6.8), flow rate, 0.3 mL/min. BCA Protein Assay Kit (Thermo Fisher Scientific) with bovine gamma globulin standard was used to determine the conjugate concentrations.

Cell lines were grown at 37°C in 5% CO_2_ in RPMI-1640 ([MDA-MB231 (human breast adenocarcinoma)]), RPMI-1640 + 0.1 mmol/L NEAA + 1 mmol/L sodium pyruvate (MKN-45 [human gastric carcinoma]) and RPMI-1640 ATCC modified with10 mmol/L HEPES, 4500 mg/L glucose (HCC-827 [human non-small cell lung carcinoma]). All media were supplemented with 10% fetal bovine serum, 2 mmol/L l-glutamine and Pen/Strep/Amphotericin B.

### Synthesis of DFO-PD-L1 mAb

The bifunctional chelating agent *p*-isothiocyanatobenzyl-desferrioxamine (DFO-Bz-NCS) was conjugated with lysine residues of PD-L1 mAb to produce DFO-PD-L1 mAb following the literature method.^24^ Briefly, PD-L1 mAb (10 mg/mL) was buffer exchanged into 0.1 mol/L NaHCO_3_ (containing 0.9% NaCl, pH 8.9), concentrated to 5 mg/mL, and treated with a 5-fold molar excess of DFO-Bz-NCS (5 mg/mL in DMSO). The mixture was gently rocked at 37°C for 75 minutes before stopping the conjugation reaction by the addition of 1 mol/L Tris (to a final concentration of 12-15 mmol/L). The DFO-PD-L1 mAb conjugate was purified twice by 2 PD-10 columns (spin protocol) into 0.25 mol/L sodium acetate (pH 5.5). Purity of PD-L1 mAb and DFO-PD-L1 mAb conjugates were determined by HPLC using size exclusion column (SE-HPLC). The concentration was measured using bicinchoninic acid assay (BCA). The number of chelators per PD-L1 mAb was determined using 25 mmol/L nonradioactive zirconium choloride mixed with a trace amount of zirconium-89 following a literature method.^25^

### Synthesis of [^89^Zr]Zr-DFO-PD-L1-mAb

[^89^Zr]Zr-DFO-PD-L1 mAb was prepared according to the method described in the literature with minor modifications.^24^ Briefly, zirconium-89 (74-111 MBq) in 1 mol/L oxalic acid was neutralized with Na_2_CO_3_ (2 mol/L). The reaction was incubated for 3 minutes. HEPES buffer (0.5 mol/L, 50 µL, pH 7.1-7.3) and gentisic acid solution (5 mg/mL, 20 µL, pH 6.5) were added to the reaction and mixed. The pH of the reaction was adjusted between 7.3 to 7.5 with Na_2_CO_3_ (2 mol/L)_._ DFO-PD-L1 mAb in 0.25 mol/L sodium acetate (0.5 mg, 4.8-5.2 mg/mL) was added, and the pH was readjusted between 7.3 to 7.5 with Na_2_CO_3_ (2 mol/L). The reaction was incubated for 1 hour at room temperature before challenging with DTPA (1 mol/L, 2 µL, pH 7) for an additional 10 minutes. The progress of the reaction was monitored by iTLC. The iTLC papers were developed with 20 mmol/L citric buffer (pH 5) and read in an Eckert & Ziegler TLC scanner (B-AR2000 -1; *R_f_* = 0.0-0.1, 96%-98% conversion). The radiolabeled conjugate was purified by PD-10 column using 0.9% NaCl (pH 7). The molar activity and the purity of the radiolabeled conjugate were determined by HPLC (*t_R_* = 9.5 minutes) using size exclusion column. Human serum albumin (20% w/v solution) was added to the radiolabeled conjugate to obtain a final concentration of 1%. The stability of the radiolabeled conjugate in whole human serum at 37°C was assessed by SE-HPLC up to 7 days (supporting information, Figure S2).

### In Vitro Studies

Saturation binding studies were performed to determine the K_d_ and B_max_ using plated cells (MDA-MB231; 2-10 × 10^5^cells/well) or cells in tubes (HCC-827; 2-10x10^5^cells/tube) to which increasing concentrations of [^89^Zr]Zr-DFO-PD-L1 mAb (0.15-52 nmol/L) were added to duplicate tubes; nonspecific binding was determined by adding unlabeled PD-L1 mAb (10^−6^ mol/L) to another set of duplicates. For competition studies, [^89^Zr]Zr-DFO-PD-L1 mAb at a single concentration (0.15-2.9 nmol/L) and increasing concentrations (0-1000 nmol/L) of competitors (PD-L1mAb) were added to MDA-MB231 or HCC-827. After incubation (2 hours, 4°C), the cell bound radiolabeled PD-L1mAb was separated from free antibody: (1) plated cells were washed with phosphate buffered saline (PBS), treated with trypsin, and collected in vials or (2) cells in tubes were pelleted by centrifugation, washed twice (PBS), and supernatants removed. The cell bound radioactivity for these samples was determined by γ counting (Perkin Elmer 2480 Wizard3, Shelton, Connecticut). From the saturation studies, the K_d_ and B_max_ were determined from 6 to 8 concentrations of radiolabeled PD-L1 mAb and analyzed using nonlinear regression curve fitting (one-site specific binding); from the competition studies, K_i_ was determined from 8 competitor concentrations (PRISM [version 5.04 Windows], GraphPad software, San Diego, California].

The biological molar activity or immunoreactive fraction (% immunoreactivity) of the [^89^Zr]Zr-DFO-PD-L1 mAb was determined by 2 methods. Briefly, in a modified method described by Lindmo et al, immunoreactivity of the radiolabeled conjugate was calculated by extrapolation to infinite antigen excess conditions.^26,27^ MDA-MB231 or HCC-827 cells were plated or aliquoted in duplicate tubes at 6 concentrations (1-40 × 10^6^ cells/mL) to which a constant concentration of [^89^Zr]Zr-DFO-PD-L1 mAb (0.15-0.40 nmol/L) was added. Nonspecific binding was determined by adding unlabeled PD-L1 mAb (10^−6^ mol/L) to another set of duplicates. All assay tubes were then incubated at 37°C for 1 hour, and the procedures performed to remove the free antibody were as described above. For the other method described by Morris, the percentage of immunoreactivity was determined by a self-displacement method derived from a radiolabeled PD-L1 mAb saturation curve and competition curve using as the competitor, unlabeled PD-L1 mAb as described earlier.^28^

### Mouse Tumor Models

Athymic female nude mice (Ncr-nu/nu, NCI-Frederick, Maryland) were injected subcutaneously in the right thigh with either MDA-MB231 (2-5 × 10^6^) in PBS:30% matrigel. All animal studies were performed in accordance with NIH Guidelines for the Care and Use of Laboratory Animals using IACUC-approved protocols.

### Biodistribution Studies

Tumor-bearing mice (tumor weights: 0.1-0.8 g) were injected while awake via the tail vein with [^89^Zr]Zr-DFO-PD-L1 mAb [0.37-0.74 MBq (10-20 µCi), 8-15 pmol] and euthanized (via CO_2_ inhalation) at selected times. Blood samples and tissues were excised from each animal, weighed, and radioactivity content was determined (Perkin Elmer 2480 Wizard3). Radioactivity content in the blood and each tissue was expressed as % injected dose per gram of tissue [%ID/g; (1)] and then normalized for body weight to a 20 g mouse (2):

(1) %ID/g = [(counts per minute (cpm) _tissue_) / (tissue weight (g))] / [cpm _total injected dose_] × 100

(2) % ID/g **_(_**_normalized to a 20 g mouse**)**_ = (%ID/g) × [(body weight)/(20 g)]

Statistical analysis of the differences between the 2 groups was done using the Student *t* test with *P* < .05 as significant (GraphPad In Stat 3 for Windows).

### PET/CT Imaging Studies

Tumor-bearing mice were anesthetized with isoflurane/O_2_ (1.5%-3% v/v) and imaged at various times after intravenous injection of [^89^Zr]Zr-DFO-PD-L1 mAb (2.6-3.7 MBq [70-100 µCi], 80-110 pmol). Whole-body static PET images were obtained at 2 bed positions (FOV = 2.0 cm, total imaging time: 10 minutes) followed by CT images (2 bed positions, 10 minutes) using the BioPET (Bioscan Inc., Washington, District of Columbia). The images were reconstructed by a 3-dimensional ordered subsets expectation maximum (3D-OSEM).

### Human PD-L1 Dosimetry Estimation

Human dosimetry estimates extrapolated from the mouse biodistribution studies were calculated using OLINDA V1.1 (Vanderbilt University, Tennessee) with mouse to human fractional organ extrapolation of the mean residence times of the ligand measured by the biodistribution described earlier. The %ID/g values (determined from the biodistribution studies described above) for a set of organs determined over a 7-day time course were used to extrapolate human dosimetry in the same organs. The whole organ was dissected from the carcass and counted to measure the organ radioactive content. For the bone, skin, muscles, and blood samples, a sample was dissected, weighed, and counted in the γ counter. Because of the relatively small uptake in the skin, muscle, and blood, these tissues were not included in the kinetic input form of the OLINDA dosimetry estimation software.

Biodistribution data showed radioconjugate uptake in the mouse skeleton above background. To account for this, a special case was made for bone dosimetry estimation. Instead of including the bone activity in the body remainder volume, the whole bone activity was estimated and entered into the trabecula bone input field in OLINDA. The bone tissue %ID/g and the %ID/organ were estimated using a murine bone fraction model of 53.3 (g/kg).^29^ This calculation gives an estimate bone mass of 1.07 g for a 20 g mouse. The %ID/g at each time point for each mouse was multiplied by the bone mass fractional estimate for each mouse.

From the %ID/organ, time–activity curves (TAC) were generated from PET images, and residence times were calculated in units of hours. Between the time points of the TAC, a trapezoidal model was used to estimate the area under the curve. For the last time point, an exponential decay curve with the half-life of zirconium-89 was used to extrapolate the tail of the TAC. Since the %ID/organ of the whole intestine was measured (including the contents), the absorbed activity between the large and small intestine was determined using the MOBY fractional mass model for a 25 g mouse.^30^ The result was that 75% of the activity was assigned to the small intestine and 25% to the large intestine. To further separate out the large intestine into upper large intestine and lower large intestine, the ICRP 80 standard of 57% contributes to the upper large intestine, and the remaining 43% was assigned to the lower large intestine.

### Histopathology and Immunohistochemistry

All tissues were routinely processed and sectioned at 5 µm for automated hematoxylin and eosin (H&E) staining and digitized with an Aperio ScanScope XT (Leica) at 200× in a single z-plane. Slides and digital images were reviewed by a board-certified veterinary pathologist.

Immunohistochemistry (IHC) staining was performed on Leica Biosystems BondMax autostainer with the following conditions: heat-induced epitope retrieval for 20 minutes and human-specific PD-L1 (Thermo, Catalog # PA5-28115) dilution 1:250 for 30 minutes. Positive control tissues included human thymus. Negative controls included isotype control reagents substituted for the primary antibody. For mouse-specific PD-L1, the following conditions were used: heat-induced epitope retrieval with Decloak Citrate for 10 minutes, mouse-specific PD-L1 (R&D Systems, AF1019) at 1:500 dilution overnight at 4°C. Positive controls included mouse spleen and negative controls included isotype control reagents substituted for the primary antibody. Slides were digitized with an Aperio ScanScope XT (Leica) at 200X in a single z-plane. Slides and digital images were reviewed by a board-certified veterinary pathologist and staining was quantified using automated image analysis algorithms from which H scores were determined. The intensity of staining was evaluated according to the following scale: 0, no staining; 1, weak staining; 2, moderate staining; and 3, strong staining. The proportion of all cells (tumor, spleen, or lymph node) found to express PD-L1 was determined and then multiplied by the staining intensity score to obtain a final semiquantitative *H* score (maximum value of 300 corresponding to 100% of cells positive for PD-L1 with an overall staining intensity score of 3).

## Results

### Radiochemistry

The DFO-PD-L1 mAb conjugate was prepared using a 5-fold molar excesses of DFO-BZ-NCS. HPLC chromatogram (size exclusion) at 280 nm indicated no aggregation of protein due to the conjugation reaction. The radiotitration assays revealed an average of 1.4 ± 0.2 DFO chelator per mAb molecule. Radiolabeling reactions with zirconium-89 proceeded at room temperature, and the isolated radiochemical yields were in the range of 80% to 95% (n = 30). The molar activities of the radioimmunoconjugates were 22,200 to 70,300 MBq/µmol (n = 30) with >95% radiochemical purity as confirmed by SE-HPLC (supporting information, Supplementary Figure S1). SE-HPLC chromatogram revealed the slow decomposition (supporting information, Supplementary Figure S2) of [^89^Zr]Zr-DFO-PD-L1 mAb at 37°C (80% intact after 7 days).

### In Vitro Cell Binding Studies

**Figure 1.**
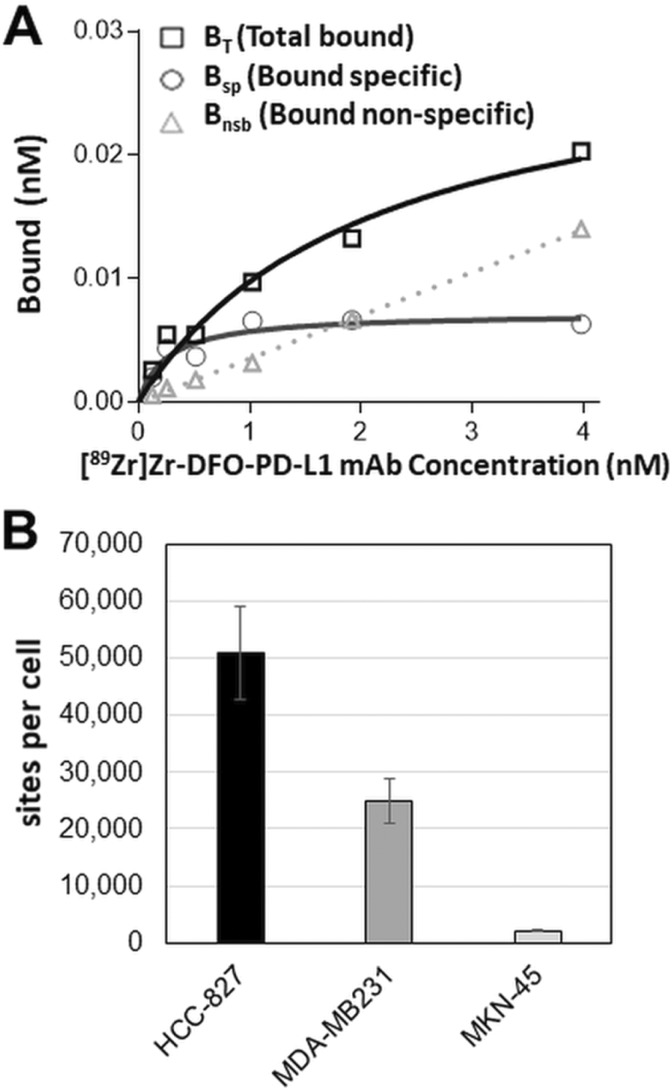
(A) Representative plot from an in vitro [^89^Zr]Zr-DFO-PD-L1 mAb saturation binding assay using MDA-MB231 cells with each point representing the average of duplicates; K_d_ = 0.29 ± 0.08 nM; B_max_ = 0.0736 ± 0.0072 nmol/L (3.71 × 10^4^ sites/cell); B_non-specific_ determined in the presence of 10^−6^ mol/L avelumab. (B) PD-L1 concentrations (B_max_) in HCC-827, MDA-MB231 and MKN-45 cancer cell lines determined from in vitro saturation binding assays; each bar represents the mean ± standard error (SE); n = 7 (HCC-827, MDA-MB231) or n = 3 (MKN-45).

In saturation binding studies, [^89^Zr]Zr-DFO-PD-L1 mAb exhibited high specific binding and affinity for PD-L1 with a K_d_ of 0.392 ± 0.0481 nmol/L (n = 7) in both MDA-MB231 (moderate/low PD-L1 expression) and HCC-827 cells (moderate; Figure 1A). HCC-827 cells had higher PD-L1 concentrations (B_max_ = 509 174 ± 8196 sites per cell; n = 7) than MDA-MB-231 cells (B_max_ = 24 973 ± 3940 sites per cell; n = 7; Figure 1B). In similar studies with MKN-45 cells PD-L1 concentrations (B_max_ = 2155 sites per cell [K_d_ = 0.55 nmol/L]) were 25- to 12-fold lower than HCC-827 and MDA-MB231 cells, respectively, with the majority of the bound [^89^Zr]Zr-DFO- PD-L1 mAb representing nonspecific binding (52%-83%; Figure 1B; supporting information, Supplementary Figure S3).

**Figure 2.**
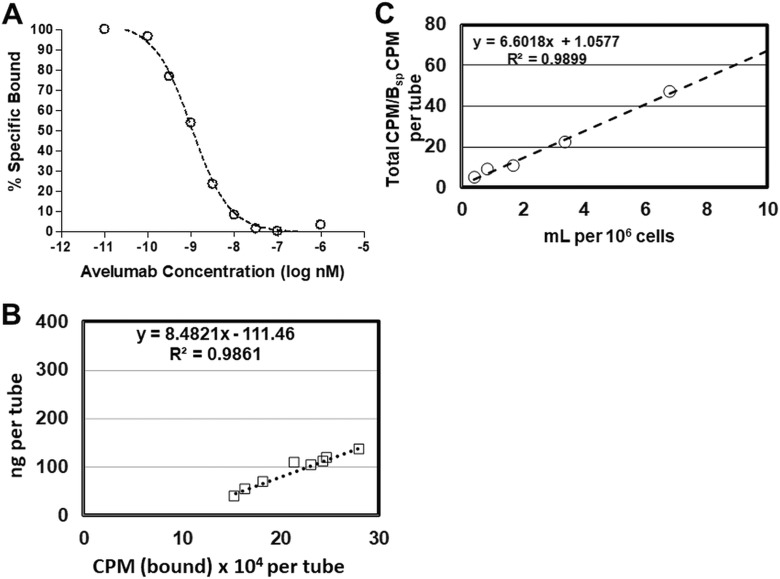
(A) Representative plot from an in vitro [^89^Zr]Zr-DFO-PD-L1 mAb competition-binding assay using avelumab [self-displacement (Morris method)] with MDA-MB231 cells. Each point (average of duplicates) represents % specific bound; K_i_ = 0.569 ± 0.07 nmol/L. Determination of the immunoreactivity fraction (%Immunoreactivity) of [^89^Zr]Zr-DFO-PD-L1 mAb from the same batch by: (B) Morris Method: Representative plot (linear regression curve fit), %Immunoreactivity = 94%; (C) Lindmo Method: Representative plot (linear regression curve fit), %Immunoreactivity = 95%.

The K_i_ of the unlabeled PD-L1 mAb (avelumab), 0.43 ± 0.048 nmol/L (n = 7), compared favorably with the K_d_ of [^89^Zr]Zr-DFO-PD-L1 mAb denoting that the conjugation with desferrioxamine (DFO) and radiolabeling with zirconium-89 had not altered the high-affinity binding to PD-L1 (Figure 2A). The biologically active fraction or immunoreactive fraction (% immunoreactivity) of the [^89^Zr]Zr-DFO-PD-L1 mAb determined by the Lindmo antigen excess method and Morris self-displacement method was 91.0% ± 3.4% (n = 6 batches) and 92.0% ± 3.4% (n = 6 batches), respectively (Figure 2B and C). Together these results would indicate that the biological activity of [^89^Zr]Zr-DFO-PD-L1 mAb was highly retained and reproducible across batches.

These in vitro results indicate that [^89^Zr]Zr-DFO-PD-L1 mAb would be appropriate for in vivo tumor imaging with low to moderate PD-L1 expression (>20 000 receptors per cell) as was observed with the HCC-827 and MDA-MB231 cells, whereas tumors with lower PD-L1 expression (<2200 sites per cell) as in the case of MKN-45 tumors would not be clearly discernable.

### In Vivo Biodistribution Studies

**Figure 3.**
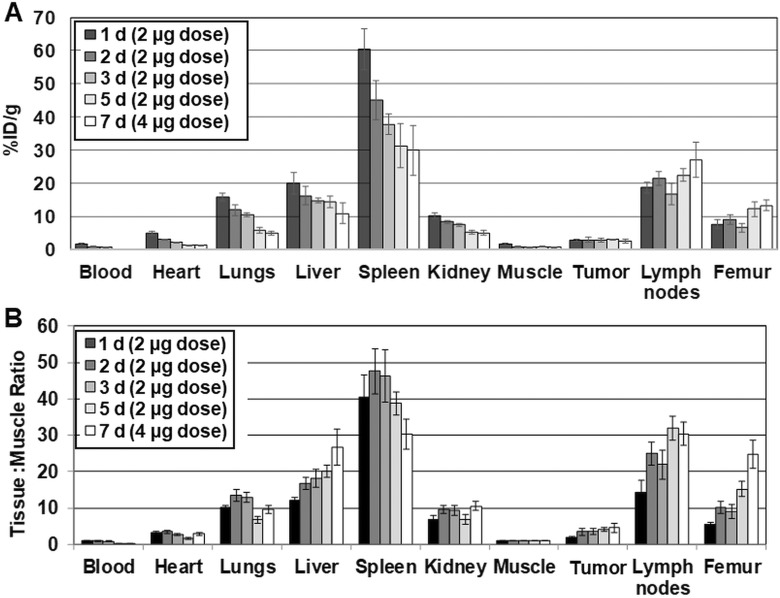
(A) Biodistribution [%ID/g (normalized to 20 g mouse)] of [^89^Zr]Zr-DFO-PD-L1 mAb in MDA-MB231 xenografts from 1 to 7 days. Each bar represents the mean %ID/g ± standard deviation (SD) of [^89^Zr]Zr-DFO-PD-L1 mAb (n = 5, 6 each time point); (B) Tissue (%ID/g) to muscle (%ID/g) ratios of [^89^Zr]Zr-DFO-PD-L1 mAb in MDA-MB231 xenografts from 1 to 7 days. Each bar in the graph represents the mean ratio ± SD of [^89^Zr]Zr-DFO-PD-L1 mAb (n = 5, 6 each time point).

The biodistribution of [^89^Zr]Zr-DFO-PD-L1 mAb was determined in MDA-MB231 xenografts, at 1, 2, 3, 5 and 7 days postinjection (Figure 3A and 3B). [^89^Zr]Zr-DFO-PD-L1 mAb distributed rapidly and cleared from nontarget tissues except for the femur over the 7-day time course (Figure 3A). The highest tissue uptakes (%ID/g) were observed in the spleen and lymph nodes at all times indicating the presence of PD-L1+cell populations (Table 1). This finding may be expected, since the PD-L1 mAb is able to recognize murine PD-L1 as well as human PD-L1 (Figure 3A). [^89^Zr]Zr-DFO-PD-L1 mAb was highly retained in MDA-MB231 tumors from day 1 (2.8%ID/g) to day 5 (3.0%ID/g) with a 13% loss occurring at day 7 (2.4%ID/g) compared to day 1. The lower MDA-MB231 tumor uptake indicates lower levels of PD-L1+ compared to the spleen and lymph nodes which agrees with the known modest PD-L1 expression levels in this cell line determined in vitro. Femur uptakes (%ID/g) increased 1.7-fold from day 1 (7.6%ID/g) to day 7 (13.2%ID/g) likely indicating loss of zirconium-89 from the DFO chelate that would be expected to localize in the bone (Table 1; Figure 3A).^31,32^ The highest tissue to muscle ratios (T: M) occurred in the spleen at day 2 (48:1) and day 3 (46:1) with decreases in T: Ms observed at day 5 (39:1) and day 7 (30:1; Figure 3B; Table 1). In contrast, the T: M ratios of lymph nodes and tumors were either unchanged or steadily increased over the 7-day imaging time course (Table 1; Figure 3B). The increases observed in the T: M ratios of these PD-L1+ tissues are most likely the result of an increased rate of clearance from the muscle rather than increased [^89^Zr]Zr-DFO-PD-L1 mAb uptake. For the most part, the minimal changes in the T: M ratios in these PD-L1+ tissues over the 7-day time course likely indicate increased retention and decreased clearance of [^89^Zr]Zr-DFO-PD-L1 mAb due to high affinity binding to PD-L1.

**Table 1.** Biodistribution (%ID/g) of [^89^Zr]Zr-DFO-PD-L1 mAb in MDA-MB231 Tumor Mouse Xenografts Over a 7-Day Time Course.

	Tissue Uptakes (%ID/g) [mean %ID/g, (SD); n = 5,6)]				
Time post injection (days)	1 d^a^	2 d^a^	3 d^a^	5 d^a^	7 d^b^
Blood	1.59 (0.30)	0.90 (0.15)	0.70 (0.13)	0.27 (0.54)	0.13 (0.02)
Heart	5.03 (0.57)	3.01 (0.20)	2.21 (0.13)	1.35 (0.15)	1.23 (0.15)
Lungs	15.87 (1.11)	11.86 (1.61)	10.54 (0.51)	5.77 (0.84)	4.94 (0.57)
Liver	20.08 (3.25)	16.22 (2.84)	14.73 (0.76)	14.36 (1.91)	10.93 (3.04)
Spleen	60.41 (6.23)	45.06 (5.92)	37.72 (3.11)	31.24 (6.62)	29.90 (7.58)
Kidney	10.26 (1.00)	8.43 (0.45)	7.53 (0.43)	5.35 (0.59)	5.06 (0.81)
Muscle	1.63 (0.31)	0.89 (0.09)	0.83 (0.10)	0.81 (0.15)	0.66 (0.19)
Tumor	2.79 (0.30)	2.99 (0.65)	2.93 (0.54)	3.03 (0.20)	2.44 (0.58)
Lymph nodes	18.64 (1.60)	21.55 (2.11)	16.68 (3.26)	22.40 (1.85)	27.03 (5.38)
Femur	7.60 (1.34)	9.03 (1.61)	6.60 (1.25)	12.24 (2.21)	13.22 (1.64)
	Tissue: Muscle Ratios (T: M) [mean T: M (SD); n=5,6)]				
Spleen	40.56 (5.94)	47.55 (6.18)	46.22 (7.18)	38.70 (3.20)	30.32 (4.10)
MDA-MB231 tumor	1.84 (0.20)	3.41 (0.90)	3.55 (0.71)	4.07 (0.50)	4.60 (1.21)
Lymph nodes	14.34 (3.30)	24.90 (3.08)	21.95 (4.05)	31.97 (3.21)	30.40 (3.18)
Femur	5.49 (0.57)	10.17 (1.62)	9.07 (2.03)	15.24 (1.98)	24.76 (3.86)

^a^ 2 µg mass associated with [^89^Zr]Zr-DFO-PD-L1 mAb injected dose.

^b^ 4 µg mass associated with [^89^Zr]Zr-DFO-PD-L1 mAb injected dose.

**Figure 4.**
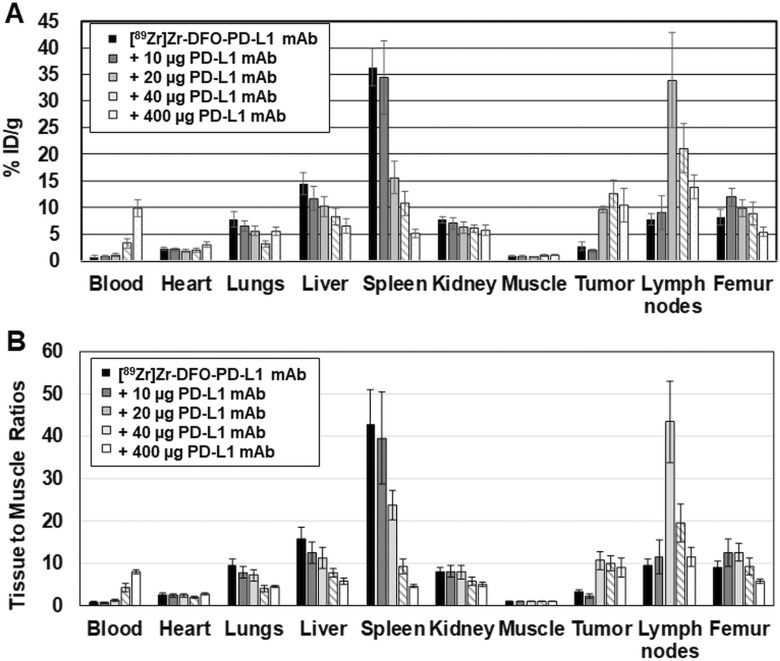
(A) Biodistribution (%ID/g) of [^89^Zr]Zr-DFO-PD-L1 mAb in MDA-MB231 xenografts 3 days after receiving coinjections of [^89^Zr]Zr-DFO-PD-L1 mAb (∼2 μg) + unlabeled PDL1 mAb (0, 10, 20, 40 and 400 μg). Each bar represents the mean %ID/g ± standard deviation (SD) of [^89^Zr]Zr-DFO-PD-L1 mAb at each dose of unlabeled PD-L1 mAb (n = 5, 6 for each group). (B) [^89^Zr]Zr-DFO-PD-L1 mAb tissue (%ID/g) to muscle (%ID/g) ratios in MDA-MB231 xenografts 3 d after receiving coinjections of [^89^Zr]Zr-DFO-PD-L1 mAb (∼2 μg) + unlabeled PDL1 mAb (0, 10, 20, 40 and 400 μg). Each bar represents the mean ratio ± SD of [^89^Zr]Zr-DFO-PD-L1 mAb at each dose of unlabeled PD-L1 mAb (n = 5, 6 for each group).

Additional biodistribution studies with MDA-MB231 xenografts were performed at 3 days after injection in which mice received escalating doses of PD-L1 mAb (10, 20, 40 or 400 µg) co-injected with [^89^Zr]Zr-DFO-PD-L1 mAb (2 µg; Figure 4A and 4B; Table 2). Spleen uptakes (%ID/g) of [^89^Zr]Zr-DFO-PD-L1 mAb were decreased as the dose of PD-L1 mAb increased indicating a dose-dependent displacement of [^89^Zr]Zr-DFO-PD-L1 mAb from the spleen. Significant decreases of 2.3-, 3.4-, and 7.1-fold were observed in the spleens of mice groups administered with PD-L1 mAb doses of 20, 40, and 400 µg, respectively, compared to the [^89^Zr]Zr-DFO-PD-L1 mAb (2 µg) only group (Figure 4A; Table 2). In contrast, uptakes (%ID/g) in lymph nodes and MDA-MB231 tumors increased significantly 2- to 5-fold at PD-L1 mAb doses of 20, 40, and 400 µg compared to the [^89^Zr]Zr-DFO-PD-L1 mAb (2 µg) only group (Figure 4A; Table 2). Similarly, the concentration of [^89^Zr]Zr-DFO-PD-L1 mAb in the blood increased, with increasing doses of PD-L1mAb with a 12.7-fold increase occurring in the 400 µg dosed group indicating that the increased mAb dose had affected the [^89^Zr]Zr-DFO-PD-L1 mAb input function (Figure 4A; Table 2). The T: M ratios were determined which would normalize these effects and provide a more accurate measure of the effects of increased mAb doses on the targeting of [^89^Zr]Zr-DFO-PD-L1 mAb (Figure 4B; Table 3). [^89^Zr]Zr-DFO-PD-L1 mAb uptake was significantly blocked in a dose-dependent manner in the spleen with decreases in T: M ratios of 44%, 78%, and 90% in the 20, 40, and 400 µg groups, respectively, compared to the [^89^Zr]Zr-DFO-PD-L1 mAb only group (Table 3). These results indicate specific [^89^Zr]Zr-DFO-PD-L1 mAb binding to PD-L1+cell populations. Inversely, tumor and lymph node T: M ratios at PD-L1 mAb doses of 20 µg and 40 µg increased significantly 2- to 5-fold compared to the [^89^Zr]Zr-DFO-PD-L1 mAb-only group (Table 3). These increased tumor and lymph node T: M ratios are most likely a result of increased availability of [^89^Zr]Zr-DFO-PD-L1 mAb in the blood due to the saturation of binding sites in the spleen with unlabeled PD-L1 mAb.

**Table 2.** Effect of PD-L1 mAb dose Escalation on the Biodistribution (%ID/g) of [^89^Zr]Zr-DFO-PD-L1 mAb in MDA-MB231 Tumor Mouse Xenografts at 3 Days Post Injection.

	Tissue Uptakes (%ID/g) [mean %ID/g (SD); n = 5,6)]				
Avelumab dose (μg) [coinjected with [^89^Zr]Zr-DFO-PD-L1 mAb]	0^a^	10^a^	20^a^	40^a^	400^a^
Blood	0.77 (0.15)	0.87 (0.17)	1.06 (0.24)	3.28 (0.87)	9.78 (1.57)
Heart	2.32 (0.32)	2.09 (0.27)	1.86 (0.23)	2.02 (0.36)	3.05 (0.42)
Lungs	7.80 (1.43)	6.47 (1.02)	5.52 (0.89)	3.14 (0.58)	5.50 (0.78)
Liver	14.50 (2.07)	11.70 (2.32)	10.14 (1.83)	8.24 (1.52)	6.52 (1.33)
Spleen	36.38 (3.47)	34.45 (6.95)	15.57 (3.05)	10.75 (2.22)	5.10 (0.72)
Kidney	7.69 (0.66)	7.05 (1.10)	6.21 (1.17)	6.01 (0.74)	5.71 (0.90)
Muscle	0.91 (0.13)	0.89 (0.11)	0.79 (0.05)	1.04 (0.22)	1.07 (0.11)
Tumor	2.71 (0.75)	1.91 (0.24)	9.64 (0.65)	12.63 (2.57)	10.38 (3.15)
Lymph nodes	7.76 (1.15)	9.03 (3.09)	33.92 (8.91)	21.10 (4.66)	13.84 (2.22)
Femur	8.15 (1.50)	11.94 (1.73)	9.85 (1.55)	8.94 (2.16)	5.37 (0.85)

^a^ 2 µg mass associated with [^89^Zr]Zr-DFO-PD-L1 mAb injected dose.

**Table 3.** Effect of PD-L1 mAb Dose Escalation on Tissue: Muscle Ratios (T: M) at 3 Days Post Injection of [^89^Zr]Zr-DFO-PD-L1 mAb.

	Tissue: Muscle Ratios [mean (SD); n= 5,6)]				
Avelumab dose (μg) [coinjected with [^89^Zr]Zr-DFO-PD-L1 mAb (2 μg)]	0	10	20	40	400
Spleen	42.57 (8.43)	39.46 (10.84)	23.64^b^ (3.46)	9.20^c^ (1.77)	4.45^c^ (0.39)
Lymph nodes	9.36 (1.63)	11.34 (3.96)	43.30^c^ (9.69)	19.41^c^ (4.44)	11.46 (2.25)
Tumor	3.09 (0.60)	2.18^a^ (0.42)	10.61^c^ (2.12)	9.95^b^ (1.68)	8.87^c^ (2.28)
Femur	8.88 (1.59)	12.51 (3.28)	12.53^a^ (2.03)	9.29 (2.00)	5.64^b^ (0.45)

^a^ *P* < .05; ^b^ *P* < .01; **^c^** *P* < .001 (n = 5 or 6 per group; Student *t* test) represents a significant difference in T: M ratio of dosed group compared to [^89^Zr]Zr-DFO-PD-L1 mAb only (0 μg avelimab dose).

**Figure 5.**
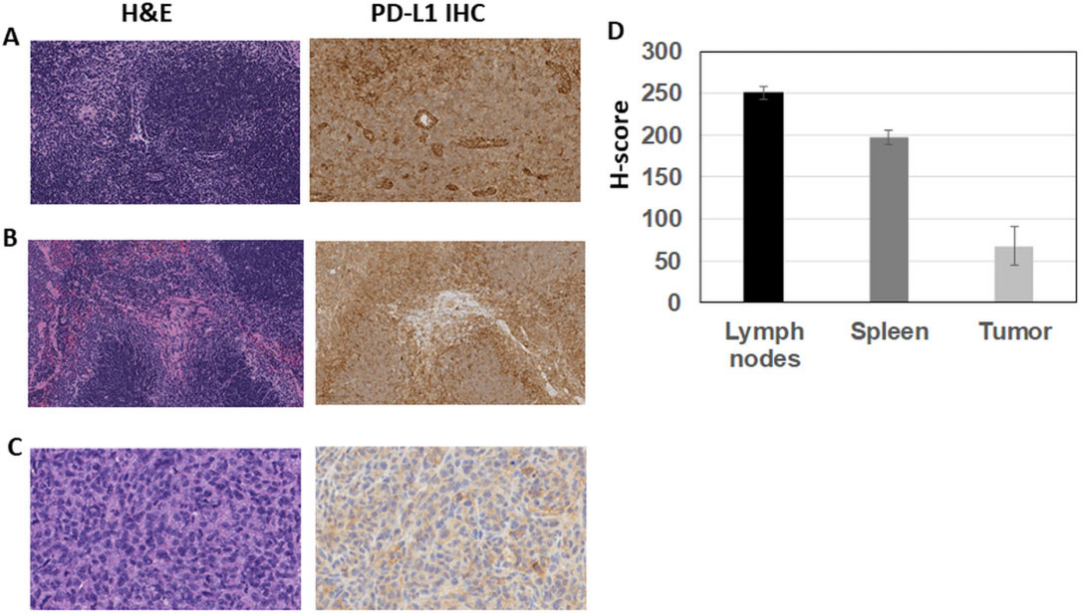
Representative images (×20 magnification) of H&E and PD-L1 IHC-stained sections (A-C): (A) Nude mouse lymph node showing normal histology (H&E) and diffuse staining for PD-L1 (mouse) with vessels and cells within the paracortical regions displaying increased staining intensity; (B) Nude mouse spleen showing normal histology (H&E) and intense membranous staining for PD-L1 (mouse) with the most intense staining at the periphery of the white pulp; (C) MDA-MB231 tumor showing a viable region (H&E necrotic regions were observed in all sections) with membranous and cytoplasmic staining for PD-L1 (human) on the tumor cells; (D) IHC quantitative analysis (staining intensity score, H-score) of PD-L1 expression levels in lymph nodes, spleen, and MDA-MB231 tumors from mouse xenografts; each bar represents the mean H-score ± standard deviation (SD; n = 3, spleen and lymph nodes; n= 6, tumors).

From these biodistribution studies, MDA-MB231 tumor, spleen, and lymph node tissues were prepared for H&E and PD-L1 IHC staining to confirm that the [^89^Zr]Zr-DFO-PD-L1 mAb uptakes in these tissues represent specific targeting to PD-L1 (Figure 5 A-C). Lymph nodes and spleen exhibited normal histology using H&E staining, whereas MDA-MB231 tumors had varying degrees of tissue necrosis which were excluded from the quantitative scoring of the PD-L1 IHC results. With IHC the highest PD-L1 expression levels (H-score) were observed in the lymph nodes (H-score =251) and spleen (H-score =198) which were 3- to 4-fold higher than the tumor (H-score = 68; Figure 5D). These IHC results correspond with the [^89^Zr]Zr-DFO-PD-L1 mAb uptakes observed in these tissues in which the spleen and lymph nodes exhibited the highest uptakes compared to the lower tumor uptakes indicating that [^89^Zr]Zr-DFO-PD-L1 mAb is able to distinguish PD-L1 expression levels in vivo.

### Micro-PET Imaging Studies

**Figure 6.**
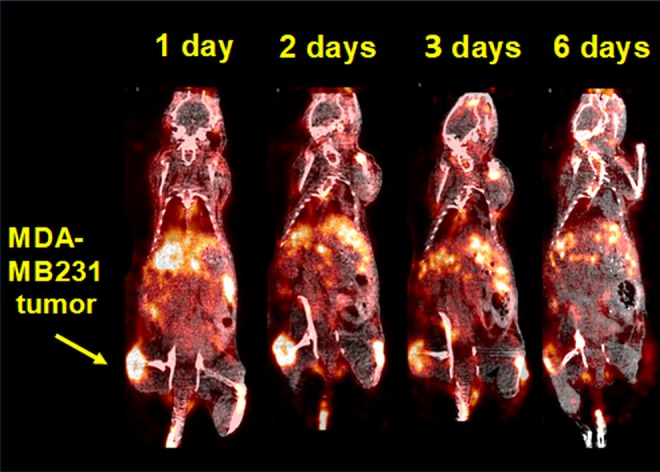
Representative coronal positron emission tomography (PET)/computed tomography (CT) images of MDA-MB231 mouse xenografts at 1, 2, 3 and 6 days postinjection (intravenous) of [^89^Zr]Zr-DFO-PD-L1 mAb [3.7 MBq (100 µCi)].

Small animal PET imaging studies were performed with MDA-MB231 xenograft mice at 1,2, 3, 6, and 7 days postinjection of [^89^Zr]Zr-DFO-PD-L1 mAb [3.7 MBq (100 µCi), associated mass ∼ 20 µg; Figure 6]. MDA-MB231 tumors and spleens were visualized in images as early as 1 day postinjection, whereas lymph nodes were more easily discerned after 3 days as radioactivity cleared from nontarget tissues (Figure 6). The MDA-MB231 T: M ratio was ∼12 at 3 days post injection which was comparable to the 20 µg PD-L1 mAb mouse group from the dose escalation biodistributions (Figure 4B; Table 3). Overall, these imaging results compared favorably with the biodistribution results and indicate that [^89^Zr]Zr-DFO-PD-L1 mAb imaging would be able to detect PD-L1+ lesions with low to moderate PD-L1 concentrations.

### Dosimetry Estimation for [^89^Zr]Zr-DFO-PD-L1 mAb

From the [^89^Zr]Zr-DFO-PD-L1 mAb biodistribution results with the MDA-MB231 xenograft mice, the radioactivity content for the organs and tissues over the imaging time course was determined and used for extrapolation for radioconjugate residence times in humans (Table 4). The 5 organs and cells that received the highest radiation absorbed dose are the spleen (1.23 mSv/MBq, 4.57 rem/mCi), osteogenic cells (0.943 mSv/MBq, 3.49 rem/mCi), red marrow (0.764 mSv/MBq, 2.83 rem/mCi), liver (0.760 mSv/MBq, 2.81 rem/mCi), and adrenals (0.578 mSv/MBq, 2.14 rem/mCi). The total body dose was 0.291 mSv/MBq or 1.08 rem/mCi, and the effective dose was 0.363 mSv/MBq or 1.34 rem/mCi.

**Table 4.** [^89^Zr]Zr-DFO- PD-L1 mAb Human Dosimetry Extrapolated from Mouse Biodistributions.^a^

Organ	Mouse Residence Time, h	Human Residence Time, h	Dose, mSv/MBq	Dose, rem/mCi
Adrenals	0.144	0.0515	0.578	2.14
Brain	0.0342	0.0412	0.179	0.663
Breasts	NA	NA	0.164	0.607
Gallbladder wall	0.00762	0.0067	0.356	1.32
LLI wall	0.257	0.0895	0.249	0.921
Small intestine	3.09	0.364	0.265	0.982
Stomach wall	0.228	0.0230	0.242	0.897
ULI wall	0.772	0.119	0.260	0.964
Heart wall	0.193	0.184	0.286	1.06
Kidneys	1.75	0.526	0.433	1.60
Liver	14.5	7.31	0.760	2.81
Lungs	0.966	2.12	0.391	1.45
Muscle	NA	NA	0.222	0.822
Ovaries	NA	NA	0.228	0.844
Pancreas	0.207	0.0337	0.351	1.30
Red marrow	NA	NA	0.764	2.83
Osteogenic cells	NA	NA	0.943	3.49
Trabecular bone	10.4	3.25	NA	NA
Skin	NA	NA	0.163	0.601
Spleen	3.44	1.79	1.23	4.57
Testes	NA	NA	0.161	0.595
Thymus	0.0479	0.0818	0.486	1.80
Thyroid	0.0556	0.0353	0.316	1.17
Urinary bladder wall	0.0245	0.0200	0.179	0.664
Uterus	NA	NA	0.210	0.775

Abbreviation: LLI, lower large intestine; ULI, upper large intestine; NA, not available.

^a^ Includes the organs used in the kinetics input form of OLINDA, the mouse residence times, the extrapolated human residence times and the dose the tissue received per unit injected activity.

## Discussion

In these preclinical studies, [^89^Zr]Zr-DFO-PD-L1 mAb exhibited specific and high-affinity PD-L1 binding in vitro and was able to distinguish between low to moderate target concentrations in PD-L1 in human cancer cell lines. In vivo [^89^Zr]Zr-DFO-PD-L1 mAb exhibited appropriate stability to be highly retained in PD-L1+ tumors as well as mouse spleen and lymph nodes which are known to have large PD-L1+ immune cell populations. These findings coupled with the normal organ dosimetry estimation results indicate that [^89^Zr]Zr-DFO-PD-L1 mAb can be considered for further clinical development as a PET imaging agent with the potential for use in selecting patients for PD-L1-targeted immunotherapeutics and treatment response monitoring.

PD-L1 has emerged as a potential biomarker for immunotherapy selection, as several PD-L1-targeted immunotherapeutics have only shown efficacy in select patient populations.^22^ Development of diagnostic agents are needed which can assess PD-L1 tumor and tumor microenvironment status. Thus far, IHC results for PD-L1 expression in pretreatment tumor biopsies do not reliably predict treatment responses.^13,18^ Further recent preclinical evidence would suggest that not just PD-L1 expression on the tumor cells is required to maintain a state of “antitumor” immunity but infiltrating lymphocytes as well.^33^ In other preclinical imaging studies in immunocompetent cancer mouse models zirconium-89- labeled anti-mouse PD-L1 mAb detected PD-L1 upregulation following irradiation which maybe an indicator of responsiveness to PD-L1-targeted therapeutics.^34^ This current situation provides an opportunity for a noninvasive molecular imaging solution for determining tumor PD-L1 expression and volume of distribution throughout the tumor and its surrounding tissues in human subjects. This imaging may provide new insights into the quantity, and distribution of PD-L1 in tumor and surrounding tissues, particularly in regard to tumor heterogeneity and temporal changes in PD-L1 expression for selection of patients and over a treatment time course.

These results are similar to previous reports in which tumor targeting and uptake of the radiolabeled antibody were affected by the total antibody protein dose.^35,36^ Chatterjee et al showed that [^111^In]In-atezolizumab uptake in human tumor xenograft mouse models was significantly improved with co-injection of 0.5 to 1.5 mg/kg of unlabeled antibody.^35^ Similarly, in other xenograft mouse studies with [^89^Zr]Zr-atezolizumab tumor uptakes were significantly increased with a protein loading dose at 15× molar excess (0.16 µCi/µg; 250 µg total protein dose) compared to the dose with no protein loading (2.5 µCi/µg; 16µg total protein dose).^37^ The increased tumor uptake of the radiolabeled antibodies could be explained by the longer blood circulation times resulting in increased levels of the radiolabeled antibody to be delivered to the tumor mass. The protein loading dose also would block specific uptake in non-tumor binding sites such as the spleen allowing a greater blood concentration of radiolabeled antibody available for tumor uptake. This interaction may explain the increased [^89^Zr]Zr-DFO-PD-L1 mAb tumor uptake observed with increased loading doses of unlabeled PD-L1 mAb in this study. The implication of this finding is that for translation to human imaging studies, an infusion of unlabeled antibody prior to radiolabeled antibody injection would improve tumor uptakes and diagnostic potential. However, this dose of avelumab would still result in serum levels below that which is expected to elicit a treatment response.

Due to the lower sub-nanomolar (K_d_∼ 0.4 nmol/L) affinity of [^89^Zr]Zr-DFO-PD-L1 mAb for human PD-L1, it would be expected to detect lower concentrations of PD-L1 compared to other nanomolar affinity probes such as [^89^Zr]Zr-atezolizumab (K_d_ ∼ 2 nmol/L, naked Ab/DFO-Ab), [^89^Zr]Zr-C4 mAb (IC_50_= 9.9 nmol/L), zirconium-89 labeled anti-PD-L1 domain Ab, KN035 (K_d_ ∼ 3 nmol/L; ∼ 80 kDa) and the radiolabeled PD-L1 specific peptide, [^64^Cu]Cu-WL12 (EC_50_ =2.9 nmol/L WL12D).^37-41^ Using the K_d_ (0.4 nmol/L) of [^89^Zr]Zr-DFO-PD-L1 mAb and the concentration of 6 nmol/L for PD-L1 on MDA-MB231 tumor cells (B_max_; determined from in vitro studies) a potential in vivo target to nontarget ratio (T: NT) of 15:1 would be predicted by applying the mathematical binding model, B_max_/K_d._ (derived from the equilibrium equation described by Scatchard)_._^42,43^ This predicted T: NT ratio compares favorably with the in vivo MDA-MB231 T: M ratio of 11:1 obtained at 20× molar excess of avelumab (20 µg; Table 3). A similar comparison can be made with [^89^Zr]Zr-atezolizumab from reported in vitro and in vivo studies with H1975 tumor cells and xenografts, respectively.^37,38^ Using the K_d_ (2 nmol/L) and the B_max_ of 6 nmol/L for the PD-L1 concentration in H1975 tumor cells (in vitro), the predicted T: NT ratio is 3:1 which is comparable to the in vivo H1975 T: M ratios ranging from 5:1 and 3:1 (at 48 hours and 72 hours, respectively, associated mass of the dose:∼ 20 µg). Therefore, these data would suggest that in humans [^89^Zr]Zr-DFO-PD-L1 mAb would be able to discern lesions with lower PD-L1 expression levels compared to other PD-L1 targeted imaging agents with lower affinity.

Although the higher affinity of [^89^Zr]Zr-DFO-PD-L1 mAb would suggest that it would be an improvement over [^89^Zr]Zr-atezolizumab in a clinical setting, the in vivo pharmacokinetics associated with these radiolabeled therapeutic mAb must also be considered. Generally, the biodistribution of [^89^Zr]Zr-DFO-PD-L1 mAb (tracer dose: 2.9 µCi/µg; total mass associated with dose: 1-2 µg) in MDA-MB231 xenografts compares with the biodistributions of [^89^Zr]Zr-atezolizumab and [^89^Zr]Zr-C4 (anti-PD-L1 recombinant IgG1 mAb) using H1975 xenografts. The tissues with the highest uptakes (%ID/g) were spleen, liver, kidney, lungs, and bone for the 3 radiotracers, although [^89^Zr]Zr-DFO-PD-L1 mAb uptake values were higher in these tissues (2- to 5-fold) at the same times.^37^ In part, the higher targeted uptakes of [^89^Zr]Zr-DFO-PD-L1 mAb in these tissues could be related to the differences associated with the radiolabeled mAb such as specific activity and the mass of mAb associated with the injected dose (represents the total mass of the radiolabeled mAb and added unlabeled mAb). For example, the highest spleen uptake (36.4%ID/g; Figure 4; Table 3) was observed with the [^89^Zr]Zr-DFO-PD-L1 mAb tracer dose that has the lowest total mass dose of 1.2 µg (highest specific activity, 2.9 µCi/µg), whereas a 2-fold decrease in the spleen uptake (15. 6%ID/g) was observed with the addition of 20 µg of unlabeled PD-L1 mAb to the tracer dose for a total mass dose of 20.1 µg (20-fold reduction in specific activity,0.15 µCi/µg; Figure 4; Table 3). Therefore, if this [^89^Zr]Zr-DFO-PD-L1 mAb biodistribution at a ∼20 µg mAb dose is compared to the biodistribution of [^89^Zr]Zr-atezolizumab also done at a similar mass dose (∼20 µg mAb), the uptake values for the spleen and lungs are now similar. However the uptake of [^89^Zr]Zr-DFO-PD-L1 mAb in the kidneys, liver, and bone are still higher (2- to 3-fold). The [^89^Zr]Zr-DFO-PD-L1 mAb decreased spleen and lung uptakes at the lower molar specific activity might be expected, since increased mass has been shown to alter radiotracer biodistributions in tissues with saturable target sites.^42^ On the other hand, the higher uptakes in the kidney liver and bone maybe more associated with clearance of the radiolabeled mAb and its metabolites rather than specific targeting. Therapeutic mAbs have been found to have complex pharmacokinetics that are influenced by their target as well as their ability to elicit immune responses. Avelumab has a biologically intact Fc receptor that is able to mediate ADCC, whereas both [^89^Zr]Zr-atezolizumab and [^89^Zr]Zr-C4 lack Fc receptor-mediated binding.^20,23,44^ In addition, the immunodeficiency status of the host mouse (strain) and other molecular properties unique to the mAb (ie, biological origin and glycosylation) have been shown to be a factor in determining their in vivo biodistribution.^45^ Moreover, in humans avelumab has been shown to have faster clearance (t_1/2_ = 3.9 − 4.1 days [10 and 20 mg/Kg doses]) than atezolizumab (t_1/2_ = 27 d) which maybe a consequence of the Fc/γ receptor-mediated binding.^46,47^ Clearance of mAb is usually biexponential with a nonlinear phase representing the pharmacokinetics of the specific binding component and a linear phase representing clearance of the nonspecific component. This faster clearance of avelumab may explain the higher kidney, liver, and bone uptake values of [^89^Zr]Zr-DFO-PD-L1 mAb compared to [^89^Zr]Zr-atezolizumab in the mouse biodistributions, as the majority of the radioactivity in these tissues would not be expected to represent intact radiolabeled mAb but radiolabeled metabolites (Ab fragments) or in the case of bone free zirconium-89.^32^ Further, this faster clearance of avelumab would indicate that suitable target to nontarget ratios maybe achieved earlier with [^89^Zr]Zr-DFO-PD-L1 mAb than with [^89^Zr]Zr-atezolizumab; thus, PET images maybe acquired sooner after the radiotracer injection.

In ongoing clinical studies, [^89^Zr]Zr-atezolizumab PET imaging showed high uptakes in tumors, normal tissues known to have PD-L1 expression (spleen, lymphoid tissues), and other tissue regions of chronic inflammation.^32,48^ In some cases, [^89^Zr]Zr-atezolizumab PET imaging was able to identify PD-L1+tumors in patients who had been scored previously as negative by IHC. While these first clinical imaging results are encouraging, further PD-L1 immuno-PET imaging studies need to be included in clinical trials to establish the predictive value of PD-L1 expression as a reliable biomarker for distinguishing responders from nonresponders, monitoring therapeutic responses and evaluating efficacy of this new class of immunotherapeutics.^49^ Likewise the clinical translation of [^89^Zr]Zr-DFO-PD-L1 mAb would aid in determining the diagnostic and prognostic potential of PD-L1 immunoPET imaging as well as increase our understanding of PD-L1 target engagement and Fc/γ-mediated responses. Moreover, since [^89^Zr]Zr-DFO-PD-L1 mAb represents the radiolabeled version of avelumab which is currently in clinical trials, this agent could not only serve as a companion diagnostic imaging agent but aid in the drug development process by establishing dosing and then tracing in vivo tissue disposition of avelumab.^50^ Although a fluorine-18-labeled small molecule radiotracer would be more desirable for clinical applications, these zirconium-89 labeled FDA-approved therapeutic mAbs are more easily translated into the clinic making possible “proof-of-concept” PET imaging studies earlier in human patients. If these studies prove that PD-L1 has value as a predictive biomarker then this would warrant further development of small molecules or Ab fragments targeting PD-L1 labeled with shorter lived radionuclides (i.e fluorine-18) for clinical applications.^39,40,51^

In conclusion PET imaging could provide quantitative assessment of PD-L1 tumor expression in the whole tumor and its microenvironment as well as in metastatic disease sites. This noninvasive imaging method could also be useful in overcoming IHC limitations in identifying patients with PD-L1 target levels predictive of treatment response. This imaging could also potentially serve as a biomarker for response monitoring for anti-PD-L1 therapy. Validated PET imaging agents designed to detect and quantitate tumor PD-L1 expression will have clinical diagnostic and prognostic value and aid in drug development by providing an accurate assessment of tumor PD-L1 status.

## Supplemental Material

Supplemental Material, PDL1_supportingInfo_ejagoda_(1) - Immuno-PET Imaging of the Programmed Cell Death-1 Ligand (PD-L1) Using a Zirconium-89 Labeled Therapeutic Antibody, Avelumab Click here for additional data file. Supplemental Material, PDL1_supportingInfo_ejagoda_(1) for Immuno-PET Imaging of the Programmed Cell Death-1 Ligand (PD-L1) Using a Zirconium-89 Labeled Therapeutic Antibody, Avelumab by Elaine M. Jagoda, Olga Vasalatiy, Falguni Basuli, Ana Christina L. Opina, Mark R. Williams, Karen Wong, Kelly C. Lane, Steve Adler, Anita Thein Ton, Lawrence P. Szajek, Biying Xu, Donna Butcher, Elijah F. Edmondson, Rolf E. Swenson, John Greiner, James Gulley, Janet Eary and Peter L. Choyke in Molecular Imaging
