# Supplementary material for: Immuno-PET Imaging of the Programmed Cell Death-1 Ligand (PD-L1) Using a Zirconium-89 Labeled Therapeutic Antibody, Avelumab
Source: Mol Imaging. 2019 May 2;18:1536012119829986. doi: 10.1177/1536012119829986 (PMC6498777; doi:10.1177/1536012119829986)
Supplement: Supplemental Material, PDL1_supportingInfo_ejagoda_(1) - Immuno-PET Imaging of the Programmed Cell Death-1 Ligand (PD-L1) Using a Zirconium-89 Labeled Therapeutic Antibody, Avelumab [file PDL1_supportingInfo_ejagoda_(1).pdf]

Supporting Information

Immuno-PET Imaging of the Programmed Cell Death-1 Ligand (PD-L1) Using a Zirconium-89 Labeled Therapeutic -Antibody, Avelumab

Elaine M. Jagoda[1], Olga Vasalatiy[2], Falguni Basuli[2], Ana Christina L. Opina[2], Mark R. Williams[1], Karen Wong[1], Kelly C. Lane[2], Steve Adler[1], Anita Thein Ton[1], Lawrence P. Szajek[3], Biying Xu[2], Rolf E. Swenson[2], John Greiner[4], James Gulley[5], Janet Eary[6] and Peter L. Choyke[1]

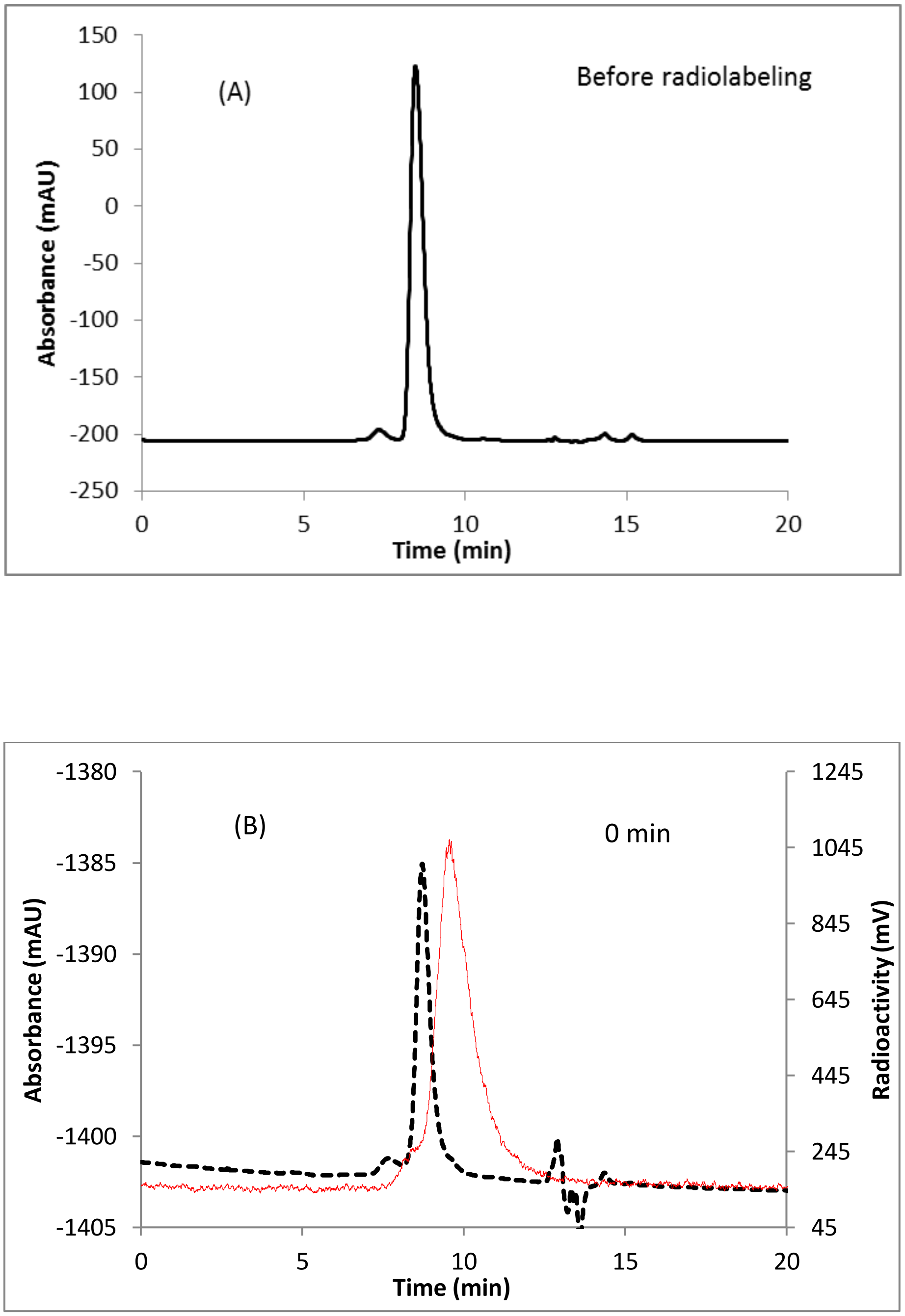

Figure S1 : HPLC chromatogram of (A) DFO-PD-L1 mAb, before radiolabeling; (B) [^{89}Zr]Zr-DFO-PD-L1 mAb. HPLC condition: eluent, 0.1 M sodium phosphate, 0.1 M sodium sulfate, 0.05% sodium azide, 10% *iso*-propyl alcohol (pH 6.8), flow rate 0.3 mL/min; black line UV detector, red line radiodetector.

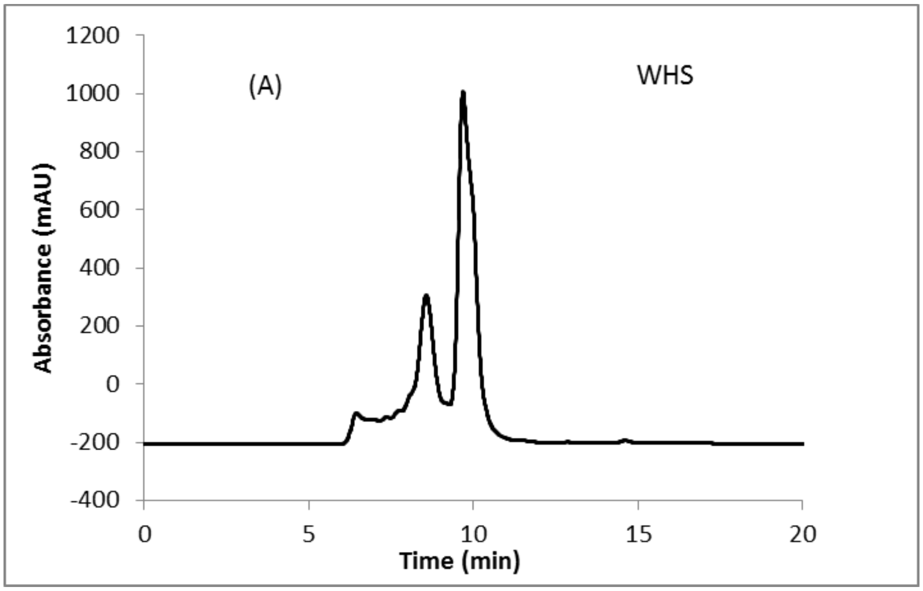

(A)
WHS
Absorbance (mAU)
Time (min)
1200
1000
800
600
400
200
0
-200
-400
0
5
10
15
20

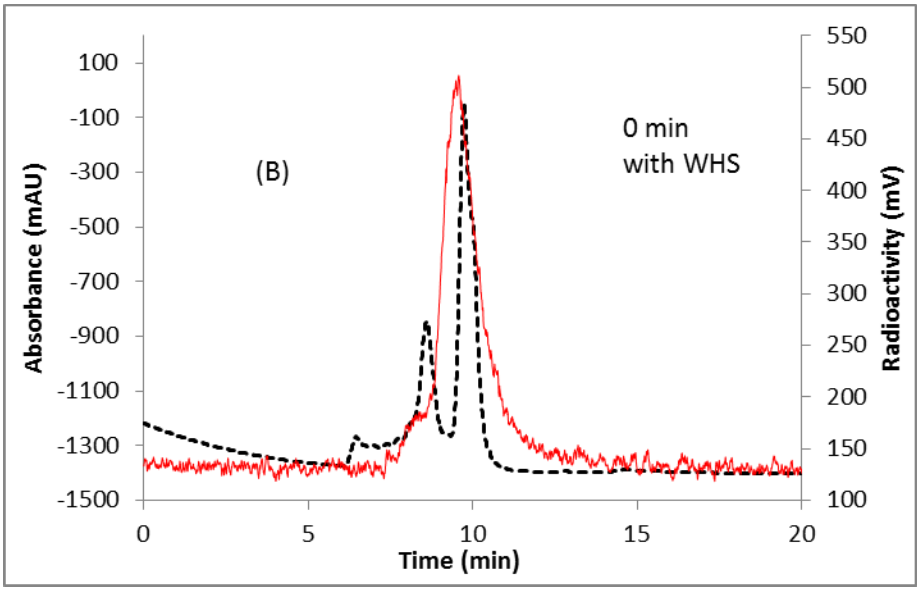

(B)
0 min
with WHS
Absorbance (mAU)
Radioactivity (mV)
Time (min)
100
-100
-300
-500
-700
-900
-1100
-1300
-1500
550
500
450
400
350
300
250
200
150
100
0
5
10
15
20

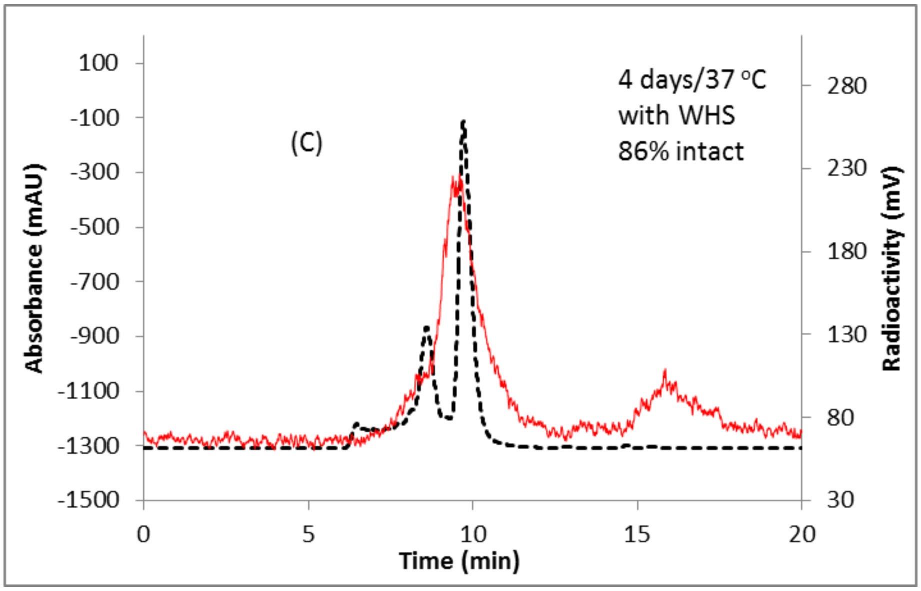

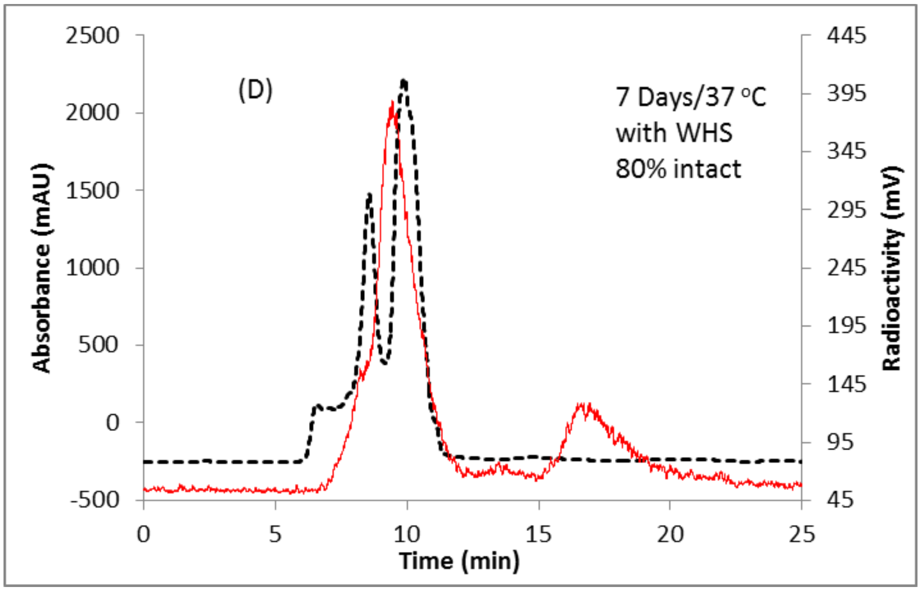

Figure S2: HPLC chromatogram of (A) whole human serum; (B) [^{89}Zr]Zr-DFO-PD-L1 mAb, 0 min; (C) [^{89}Zr]Zr-DFO-PD-L1 mAb, 4 days at 37 ºC;. (D) [^{89}Zr]Zr-DFO-PD-L1 mAb, 7 days at 37 ºC. HPLC condition: eluent, 0.1 M sodium phosphate, 0.1 M sodium sulfate, 0.05% sodium azide, 10% *iso*-propyl alcohol (pH 6.8), flow rate 0.3 mL/min; black line UV detector, red line radiodetector.

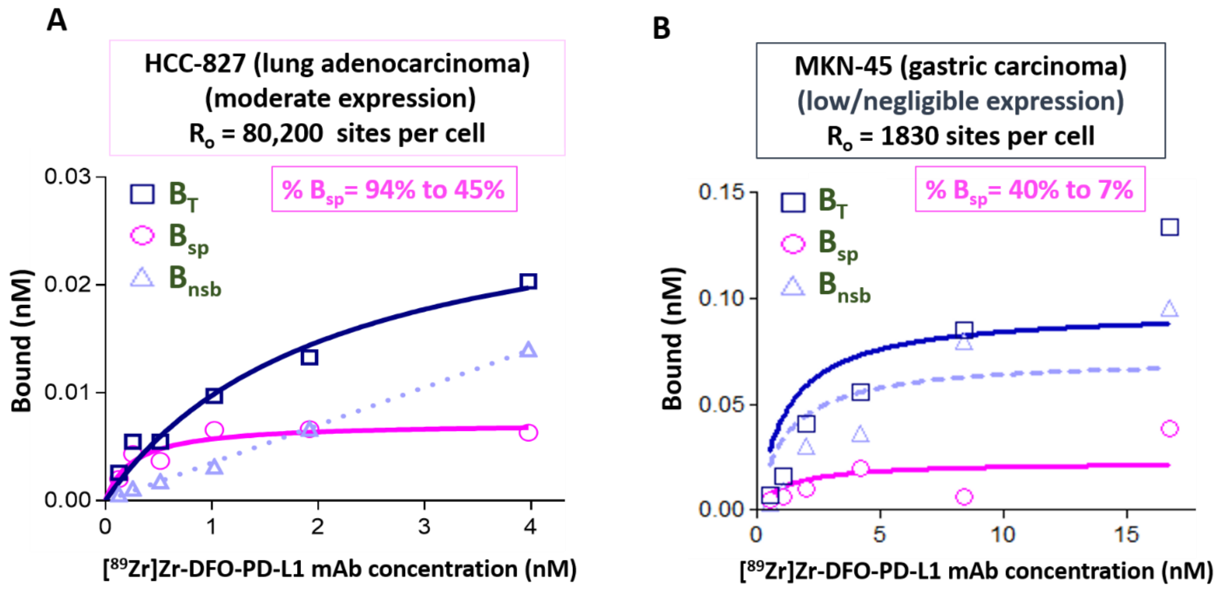

Figure S3: A) Representative in vitro [^{89}Zr]Zr-DFO-PD-L1 mAb saturation binding assay using HCC- 827 cells with each point representing the average of duplicates; **B)** Representative in vitro [^{89}Zr]Zr-DFO-PD-L1 mAb saturation binding assay using HCC- 827 cells with each point representing the average of duplicates; $B_{T=}$ B_{Total}; $B_{sp=}$ $B_{specific}$; $B_{nsb\,=}$ $B_{non\text{-}specific}$ (determined in the presence of 10^{-6} M avelumab)
